# Heterozygote Advantage Probably Maintains Rhesus Factor Blood Group Polymorphism: Ecological Regression Study

**DOI:** 10.1371/journal.pone.0147955

**Published:** 2016-01-26

**Authors:** Jaroslav Flegr

**Affiliations:** Division of Biology, Faculty of Science, Charles University in Prague, Prague, Czech Republic; University of Palermo, ITALY

## Abstract

Rhesus factor polymorphism has been an evolutionary enigma since its discovery in 1939. Carriers of the rarer allele should be eliminated by selection against Rhesus positive children born to Rhesus negative mothers. Here I used an ecologic regression study to test the hypothesis that Rhesus factor polymorphism is stabilized by heterozygote advantage. The study was performed in 65 countries for which the frequencies of RhD phenotypes and specific disease burden data were available. I performed multiple multivariate covariance analysis with five potential confounding variables: GDP, latitude (distance from the equator), humidity, medical care expenditure per capita and frequencies of smokers. The results showed that the burden associated with many diseases correlated with the frequencies of particular Rhesus genotypes in a country and that the direction of the relation was nearly always the opposite for the frequency of Rhesus negative homozygotes and that of Rhesus positive heterozygotes. On the population level, a Rhesus-negativity-associated burden could be compensated for by the heterozygote advantage, but for Rhesus negative subjects this burden represents a serious problem.

## Introduction

Polymorphism in the Rhesus factor, namely the existence of a large deletion in the RHD gene [1] in a substantial fraction of the human population, has been an evolutionary enigma since the discovery of this factor in the 1930’s [2–5]. Before the introduction of prophylactic treatment in 1968, the carriers of the rarer variant of the gene, namely Rhesus negative women in in a population of Rhesus positive subjects or Rhesus positive men in population of Rhesus negative subjects, had lower fitness. This is because RhD-positive children born to pre-immunized RhD-negative mothers were at a higher risk of fetal and newborn death or health impairment from the haemolytic disease. Therefore, mutants or migrants with the rarer variant of the RHD gene could not invade the population and any already existing RhD polymorphism should be unstable.

It has been suggested that this polymorphism can be stabilized when the disadvantage of carriers of the locally rarer allele is counterbalanced by higher viability of their heterozygote children or by another form of frequency-dependent selection [6]. In the past seven years, several studies have demonstrated that Rhesus positive and Rhesus negative subjects differ in resistance to the adverse effects of parasitic infections, aging, fatigue and smoking [7–13]. A recently published cross sectional study performed on a cohort of on 3,130 subjects showed numerous associations between Rh negativity and incidence of many disorders. In this study, one hundred fifty four (154) of 225 diseases/disorders were reported by at least 10 subjects. Within this subset, 31 significant associations with RhD negativity (21 positive and 10 negative) were observed [14]. A study performed on 250 blood donors has further shown that resistance to the effects of toxoplasmosis is higher in Rhesus positive heterozygotes than in Rhesus positive homozygotes and substantially higher than in Rhesus negative homozogotes [7]. This is the first direct evidence for the role of selection in favour of heterozygotes in stabilization of the RHD gene polymorphism in human populations. Such a mechanism is reminiscent of widely known situations with polymorphism in genes associated with sickle cell anaemia in geographic regions with endemic malaria [15].

RhD protein is a component of a membrane complex of which the function is not quite clear. It is most probably involved in NH_3_ transport and possibly also in CO_2_ transport [16,17]. The complex is associated with spectrin-based cytoskeleton and therefore plays an important role in maintaining the typical shape (biconcave discoid) of human erythrocytes [18]. The “European” RhD- variant of the RHD gene carries a deletion covering the entire protein-coding part of the gene [1]. Therefore, no product of this allele is synthetized in the cells of RhD negative homozygotes and the RhD is most probably substituted in the corresponding molecular complex by the related protein RhCE. Therefore, erythrocytes of RhD- and RhD+ homozygotes differ in molecular complexes on their cell membranes and probably also in their biological activities. An important difference was also observed between erythrocytes of RhD positive homozygotes and heterozygotes. About 33,560 and 17,720 D antigen sites were detected on the surfaces of an erythrocyte in RhD homozygotes and heterozygotes, respectively [18]. This suggests that the susceptibility of RhD positive homozygotes and RhD positive heterozygotes (and even more so RhD negative homozygotes) to various aberrant conditions, including various diseases, could differ dramatically. Due to the general trade-off principle, heterozygotes could be more resistant to one disease and more prone to another disease while the opposite could be true for homozygotes. Such trade-offs could explain the heterozygote advantage hypothesis and all other observed phenomena.

The frequencies of Rhesus negative subjects (and therefore also Rhesus positive heterozygotes) as well as the incidences of particular diseases and disorders vary between countries. If the protective effect of Rhesus positivity or Rhesus heterozygosity is strong enough, then the relationship between the frequencies of Rhesus negative homozygotes (and Rhesus positive heterozygotes) should correlate with the incidences of specific diseases when important confounding variables are controlled. This could be either because the incidences of particular disease influence the geographic distribution of RhD alleles, or because the differences in prevalence of particular phenotypes influence the incidences of particular diseases. Here, I have studied the correlation of disease burden estimates compiled by the WHO with the frequencies of Rhesus negative homozygotes and Rhesus positive heterozygotes in a set of 65 countries for which the data on the frequencies of Rhesus-negative individuals are available.

The specific aims of present study (the hypothesis to be tested] are:

Hypothesis 1: The frequency of Rh negative homozygotes in particular countries correlates (mostly positively) with the incidence of some health disorder in these countries
Hypothesis 2: The frequency of Rh positive heterozygotes in particular countries correlates (mostly negatively) with the incidence of some health disorder in these countries
Hypothesis 3:. The relation of the incidence of health disorders with frequency of Rh negative and Rh positive heterozygotes are mostly in opposite directions.

## Materials and Methods

### Sources of Data

The data on disease burdens were obtained from the table “Mortality and Burden of Diseases Estimates for WHO Member States in 2004,” which was published by the WHO [19] and are available at: www.who.int/evidence/bod. Because of the expected effects of the frequencies of the Rhesus factor genotypes on the age structures of the populations, the age-non-standardized disease burden data were used. The frequencies of Rhesus negative homozygotes in particular countries [20] were taken from the internet compilation by RhesusNegative.net available at http://www.Rhesusnegative.net/themission/bloodtypefrequencies/ from December, 22^nd^ of 2013 and from the monograph of Mourant [21]. The frequencies of Rhesus positive heterozygotes were calculated from the frequencies of Rhesus negative homozygotes using the Hardy-Weinberg equation. The geographical latitude and the annual mean of relative humidity for particular countries were derived using the data available at http://data.worldbank.org/indicator/NY.GDP.PCAP.CD (accessed December, 10^th^ of 2013) and http://www.climatemps.com/ (accessed April, 2^nd^ of 2013). The medical care expenditure expenses per capita were obtained from http://data.worldbank.org/indicator/SH.XPD.PCAP (accessed March, 2^nd^ of 2014). The frequencies of smokers in the populations were calculated as the arithmetic mean from men and women; data available at http://www.who.int/tobacco/mpower/mpower_report_prevalence_data_2008.pdf (accessed February, 21^st^ of 2014). All data used in this study are available as S1 Data.

### Statistical Methods

Factor analyses and a general linear model (GLM) analysis of the obtained factors were performed with Statistica v. 8.0 and all other tests with IBM SPSS v. 21. A factor analysis of the specific disease burden data (principal components method, raw Varimex rotation) was used for data reduction. When the factors were extracted from the mortality rate variables, the diseases with not enough data were excluded and the missing data were substituted with means. The Disability Adjusted Life Year (DALY) data for nearly all diseases except trypanosomiasis, Chagas disease, and onchocerciasis were available for all countries in my data set. All factors with eigenvalues > 1.0 were extracted. Type III sum of squares and models including the intercept were used in the GLM (multivariate and multiple-multivariate) analyses; for details, see [22].

## Results

### Association between Disease Burden and Frequencies of RhD Genotypes

Mortality and morbidity data for more than one hundred diseases are available in the WHO database [19]. A search for associations between Rhesus genotype and particular diseases would necessarily result in many spurious associations. Therefore, factor analysis was initially used for data reduction. Performing this analysis on 125 diseases and burden of disease categories yielded 23 factors with eigenvalues>1.0, together explaining 85% of the variability in Disability Adjusted Life Year (DALY) among 192 WHO member countries. The DALY has been defined [19] as “a health gap measure that extends the concept of potential years of life lost due to premature death and also to include equivalent years of ‘healthy’ life lost by virtue of being in a state of poor health or disability”. Next, a multiple multivariate analysis was performed with these 23 factors as dependent variables and frequencies of Rhesus negative homozygotes, Rhesus positive heterozygotes and also five potential confounding variables: GDP, latitude (distance from the equator), humidity, medical care expenditure per capita and frequencies of smokers in the population for 65 countries with the RhD genotype frequency data as independent variables. The effects of the frequency of RhD heterozygotes (*μ*^*2*^ = 0.67, *P* = 0.013), smokers (*μ*^*2*^ = 0.75, *P* = 0.001), latitude (*μ*^*2*^ = 0.71, *P* = 0.005), and humidity (*μ*^*2*^ = 0.64, *P* = 0.027) but not frequency of RhD negative homozygotes (*μ*^*2*^ = 0.60, *P* = 0.070), GDP (*μ*^*2*^ = 0.42, *P* = 0.635) and medical care expenditure (*μ*^*2*^ = 0.23, *P* = 0.991) were significant. This model explained considerable parts of variability in factors 1–3, 6–8, 11, and 22 (adjusted *R*^*2*^ = 0.372, 0.771, 0.497, 0.461, 0.197, 0.237, 0.224, and 0.296, respectively). Post hoc simple multivariate analyses showed that the frequency of Rhesus negative homozygotes correlated with five of 23 factors that together explained 17.7% of variability between different countries in DALY (Table 1). Similarly, the frequency of RhD positive heterozygotes significantly correlated with six factors that together explained 13.9% of variability between different countries in DALY. Three correlations of 23 factors with frequency of RhD positive homozygotes and one with RhD negative homozygotes remained significant even after the correction for multiple tests [23]. Moreover, the expected number of false significant results for 23 statistical tests was not 5.5 but 1.2, suggesting that most of the positive results were not simply due to multiple comparisons. In accord with the heterozygote advantage hypothesis, the regression coefficients (*B*-values) for Rhesus negative homozygotes and Rhesus positive heterozygotes went toward opposite directions whenever any of these correlations was significant.

**Table 1:** Correlations of 23 independent factors explaining 85% of variability in disease burden with the frequencies of RhD genotypes.

		RhD genotypes		Confounding variables				
		RhD negat.	RhD heteroz.	HDP	Latitude	Humidity	Medic. care	Smokers
Factor 1	Beta	-0.385	0.697	-0.527	-0.335	0.261	0.336	-0.224
(29.5%)	*P*	0.337	**0.058**	**0.053**	**0.081**	0.028*	0.187	0.157
Factor 2	Beta	-0.576	0.138	-0.063	-0.069	-0.139	-0.329	-0.168
(10.1%)	*P*	0.020*	0.528	0.695	0.545	**0.053**	**0.035**	**0.080**
Factor 3	Beta	-0.363	0.388	-0.465	0.439	0.033	-0.326	0.377
(7.5%)	*P*	0.313	0.233	**0.056**	**0.012**	0.750	0.154	**0.009**
Factor 4	Beta	-0.595	0.474	-0.265	0.331	0.138	0.050	0.063
(5.4%)	*P*	0.245	0.304	0.436	0.172	0.352	0.875	0.752
Factor 5	Beta	0.240	-0.229	-0.199	0.286	-0.096	-0.074	-0.258
(3.9%)	*P*	0.645	0.626	0.567	0.248	0.525	0.821	0.210
Factor 6	Beta	-0.122	0.996	0.463	-0.725	0.219	-0.233	0.162
(3.8%)	*P*	0.742	0.004*	**0.065**	0.000*	0.046*	0.322	0.269
Factor 7	Beta	1.593	-1.524	-0.035	0.159	-0.038	0.057	-0.268
(2.9%)	*P*	0.001*	0.000*	0.908	0.458	0.773	0.840	0.134
Factor 8	Beta	0.841	-0.985	-0.216	0.005	0.036	-0.225	-0.259
(2.6%)	*P*	**0.061**	0.016*	0.464	0.982	0.778	0.420	0.138
Factor 9	Beta	1.148	-0.962	-0.335	-0.449	-0.014	0.301	-0.092
(2.2%)	*P*	0.019*	0.029*	0.294	**0.050**	0.919	0.319	0.622
Factor 10	Beta	-0.159	**0.093**	0.148	0.017	0.206	-0.135	-0.183
(2.0%)	*P*	0.757	0.840	0.665	0.944	0.171	0.677	0.364
Factor 11	Beta	0.658	-1.184	0.160	0.309	-0.071	-0.244	-0.021
(1.8%)	*P*	0.143	0.005*	0.591	0.146	0.582	0.387	0.904
Factor 12	Beta	0.062	-0.210	-0.084	0.582	-0.162	-0.215	-0.033
(1.6%)	*P*	0.898	0.632	0.795	**0.014**	0.254	0.484	0.861
Factor 13	Beta	-1.008	0.576	0.520	0.201	-0.246	-0.112	0.141
(1.5%)	*P*	0.042*	0.193	0.113	0.384	**0.087**	0.717	0.459
Factor 14	Beta	0.634	-0.229	-0.191	-0.338	-0.027	-0.096	-0.096
(1.3%)	*P*	0.201	0.607	0.561	0.150	0.849	0.758	0.619
Factor 15	Beta	0.457	-0.128	-0.674	0.028	-0.020	0.323	-0.360
(1.3%)	*P*	0.341	0.767	**0.039**	0.903	0.887	0.287	**0.059**
Factor 16	Beta	-0.812	0.718	0.194	0.196	-0.238	-0.092	-0.008
(1.2%)	*P*	0.107	0.114	0.560	0.406	0.104	0.771	0.966
Factor 17	Beta	1.040	-0.643	0.000	-0.303	0.351	-0.064	-0.131
(1.1%)	*P*	0.033*	0.139	1.000	0.183	**0.014**	0.831	0.484
Factor 18	Beta	-0.159	0.199	-0.209	0.178	-0.186	0.217	-0.026
(1.1%)	*P*	0.758	0.669	0.545	0.466	0.219	0.506	0.897
Factor 19	Beta	-0.543	0.791	-0.054	-0.572	0.190	0.079	0.473
(1.0%)	*P*	0.269	**0.078**	0.868	**0.016**	0.185	0.798	**0.017**
Factor 20	Beta	0.032	0.086	-0.296	-0.418	0.081	0.286	0.402
(1.0%)	*P*	0.948	0.849	0.379	**0.083**	0.582	0.369	**0.045**
Factor 21	Beta	0.651	-0.314	-0.367	0.008	0.033	0.086	-0.296
(1.0%)	*P*	0.204	0.496	0.283	0.974	0.824	0.789	0.142
Factor 22	Beta	-0.761	0.382	0.651	-0.219	0.410	-0.277	0.245
(0.8%)	*P*	**0.077**	0.320	**0.025**	0.276	0.002*	0.303	0.145
Factor 23	Beta	-0.491	0.954	-0.134	-0.565	0.292	**0.097**	0.430
(0.8%)	*P*	0.303	0.030*	0.673	**0.015**	**0.038**	0.748	**0.024**

The first column shows (in parentheses) the percentages of variability between countries in the total disease burden (DALY) explained by a particular factor. The columns 3–9 show partial correlation (*Beta*) and statistical significance (*P*) of the correlations between the factors 1–23 and the frequencies of RhD- homozygotes, RhD+ heterozygotes, HDP, latitude, humidity, medical care expenditure per capita, and frequencies of smokers in the population. Significant results (*P* < 0.05) and trends (*P* < 0.10) are printed in bold. Asterisks indicate results significant in two-sided tests after the correction for multiple test. Values < 0.0005 are coded as 0.000.

### Correlation between Particular Disease Burdens and Frequencies of RhD Genotypes

**Fig 1:**
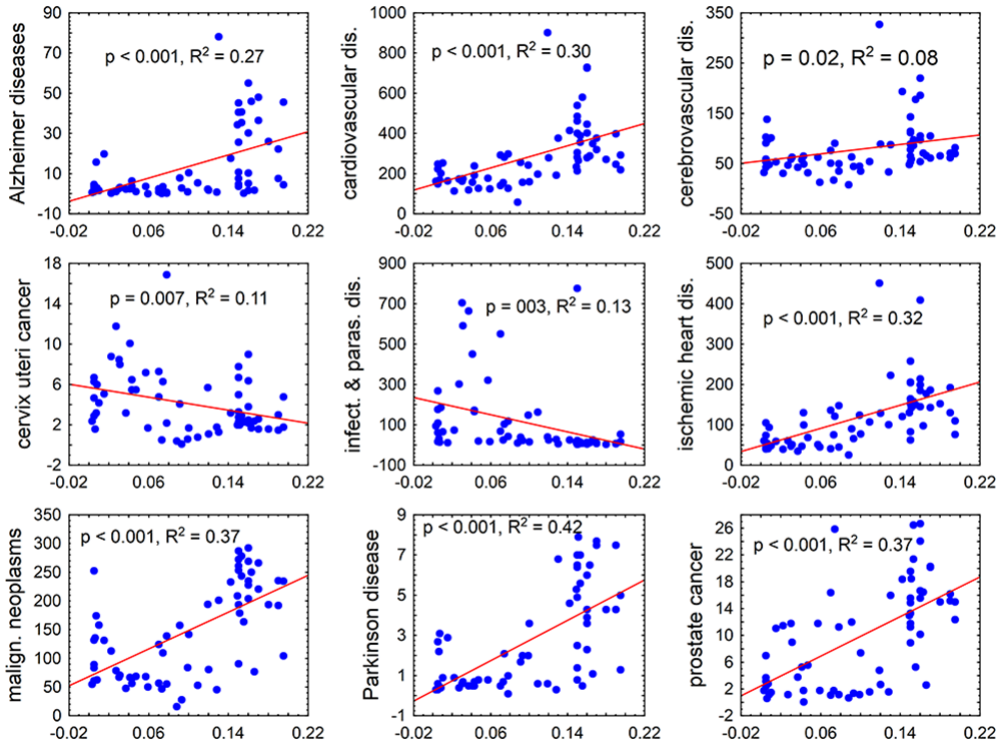
Correlation of the frequencies of Rhesus negative subjects in 65 countries with the mortality rates for nine diseases or disease categories. The x and y axes show the frequency of Rhesus negative homozygotes in the population of a country and mortality (numbers of deaths per population of 100,000), respectively. The figures represent the results of the Pearson correlation analysis, namely the level of significance (*P*) and the coefficient of determination, i.e. the fraction of a country’s variability of the specific mortality rate that can be explained by the differences within the frequencies of Rhesus negative subjects.

In the exploratory part of the study, univariate correlations analyses showed many strong associations between disease burdens measured with DALY or with incidences of deaths per population of 100,000 and the frequencies of Rhesus negative subjects or Rhesus positive heterozygotes (Fig 1). However, specific disease burdens also strongly correlated with some of the confounding variables, most often with latitude, smoking and humidity. These covariates expressed either highly significant correlation with frequency of Rhesus negative subjects and Rhesus positive heterozygotes (GDP: *R* = 0.64, *P* < 0.000001, latitude: *R* = 0.65, *P* < 0.000001, medical care expenditure per capita: *R* = 0.68, *P* < 0.000001, frequencies of smokers in the population: *R* = 0.34, *P* < 0.001; Spearman correlation), or a trend (humidity: *R* = -0.19, *P* = 0.132). Therefore, the GLM analysis was used to search for the association between Rhesus genotypes and disease burden. This was accomplished by using the frequency of Rhesus negative homozygotes and Rhesus positive heterozygotes and GDP, latitude, humidity, medical care expenses per capita and frequencies of smokers within the populations of 65 countries possessing the RhD genotype frequency data. Tables supplied by the WHO contained information regarding 121 diseases and disease categories for this subset of countries. The results presented in the Table 2 show that the frequencies of Rhesus negative subjects correlated with DALY for 21 of 121 diseases and disease categories (twelve positively and nine negatively). The frequency of Rhesus positive heterozygotes correlated with DALY for 25 of 121 diseases and disease categories (eleven positively and fourteen negatively). Similarly, the frequencies of Rhesus negative subjects correlated (all positively) with the mortality rates (incidences of deaths per population of 100,000) for 10 of 97 diseases and disease categories with mortality rate data available. The frequencies of Rhesus positive heterozygotes correlated with the mortality rates for 8 of 97 diseases and disease categories (two positively and six negatively). The expected number of false significant results for 436 statistical tests was not 65 but 22, however, due to the complicated network of correlation between incidence (or morbidity) of particular diseases (which did not exist between non-correlated factors obtained by the factor analysis) it was not possible to perform an unbiased formal correction for multiple tests (Garcia 2004) [23].

**Table 2:** Correlation between the frequencies of Rhesus factor genotypes and specific disease burden.

	mortality						DALY					
	RhD-negatives			heterozygotes			RhD-negatives			heterozygotes		
	B	p	Eta^2^	B	p	Eta^2^	B	p	Eta^2^	B	p	Eta^2^
**All Causes**	4463.26	**0.051**	0.051	-1579.49	0.120	0.120	52091.6	0.497	0.497	-14033.2	0.683	0.023
**Communicable, maternal, perinatal & nutritional conditions**	608.73	0.696	0.696	-308.57	0.659	0.659	21617.7	0.700	0.700	-6913.73	0.783	0.061
**A. Infectious and parasitic dis.**	581.09	0.509	0.509	-351.51	0.375	0.375	17081.7	0.567	0.567	-9732.23	0.468	0.114
**Tuberculosis**	-12.27	0.919	0.919	13.88	0.798	0.798	344.41	0.905	0.905	93.89	0.942	0.137
**STDs excluding HIV**	19.25	0.633	0.633	-9.06	0.603	0.603	25.15	0.983	0.983	-104.96	0.846	0.044
Syphilis	68.86	0.726	0.726	-18.29	0.731	0.731	202.15	0.832	0.832	-136.97	0.749	0.001
Chlamydia							-140.91	0.510	0.510	19.05	0.842	0.062
Gonorrhoea							-206.81	0.444	0.444	47.57	0.694	0.141
**HIV/AIDS**	365.93	0.274	0.274	-222.41	0.141	0.141	8820.72	0.336	0.336	-5910.93	0.154	0.125
**Diarrhoeal diseases**	141.75	0.605	0.605	-38.77	0.749	0.749	3421.41	0.680	0.680	-609.46	0.870	0.030
**Childhood-cluster diseases**	-18.66	0.862	0.862	4.51	0.922	0.922	-43.77	0.987	0.987	-231.72	0.847	0.030
Pertussis	-130.66	0.452	0.452	28.71	0.630	0.630	164.18	0.887	0.887	-89.30	0.864	0.022
Poliomyelitis	12.94	0.590	0.590	-23.47	0.594	0.594	-4.77	0.674	0.674	0.93	0.878	0.037
Diphtheria							24.53	0.917	0.917	-34.58	0.648	0.037
Measles	-59.71	0.766	0.766	36.99	0.526	0.526	-5962.72	0.615	0.615	2593.09	0.403	0.001
Tetanus	-145.36	0.238	0.238	27.80	0.382	0.382	-4955.75	0.130	0.130	1028.00	0.222	0.005
**Meningitis**	30.07	0.468	0.468	-14.51	0.436	0.436	790.37	0.570	0.570	-328.56	0.599	0.063
**Hepatitis B**	13.74	0.304	0.304	-7.62	0.207	0.207	235.12	0.371	0.371	-90.24	0.445	0.058
**Hepatitis C**	15.22	0.132	0.132	-8.29	**0.084**	0.084	262.78	**0.068**	0.068	-131.07	**0.055**	0.124
**Malaria**	-2608.0	0.292	0.292	664.47	0.293	0.293	-32353.1	0.302	0.302	6622.57	0.486	0.013
**Tropical-cluster diseases**	59.42	0.761	0.761	-16.67	0.818	0.818	-10929.5	0.114	0.114	3189.37	0.132	0.000
Leishmaniasis							-609.77	0.857	0.857	-144.33	0.877	0.028
Lymphatic filariasis							2425.38	0.778	0.778	-883.68	0.675	0.592
**Leprosy**	-9.98	0.828	0.828	2.37	0.847	0.847	10.23	0.961	0.961	3.39	0.958	0.134
**Dengue**							-1841.32	0.165	0.165	420.93	0.185	0.688
**Intestinal nematode infections**							-1025.99	0.544	0.544	42.68	0.930	0.000
Ascariasis							-837.39	0.624	0.624	2.60	0.996	0.011
Trichuriasis							127.63	0.746	0.746	-32.78	0.796	0.001
Hookworm dis.							513.99	0.132	0.132	-281.45	0.020*	0.004
**B. Respiratory infections**	41.00	0.914	0.914	-34.59	0.839	0.839	3311.07	0.761	0.761	-197.17	0.968	0.037
Lower respiratory infections	31.50	0.933	0.933	-30.25	0.857	0.857	3171.15	0.766	0.766	-159.78	0.973	0.040
Upper respiratory infections	11.90	0.288	0.288	-3.51	0.395	0.395	237.87	0.358	0.358	-80.69	0.486	0.022
Otitis media	14.26	0.300	0.300	-3.65	0.378	0.378	-100.69	**0.078**	0.078	44.79	**0.081**	0.033
**C. Maternal conditions**	32.59	0.675	0.675	-15.56	0.647	0.647	-91.82	0.979	0.979	225.39	0.888	0.108
**D. Perinatal conditions**	-5.35	0.987	0.987	55.82	0.699	0.699	431.32	0.974	0.974	2378.76	0.687	0.000
Prematurity and low birth weight	-30.27	0.761	0.761	34.53	0.441	0.441	-871.28	0.828	0.828	1268.44	0.482	0.002
Birth asphyxia, birth trauma	3.29	0.969	0.969	10.89	0.778	0.778	392.87	0.927	0.927	737.27	0.701	0.000
Neonatal infections, other conditions	23.23	0.873	0.873	9.76	0.881	0.881	916.91	0.859	0.859	371.32	0.873	0.001
**E. Nutritional deficiencies**	-23.46	0.694	0.694	27.27	0.312	0.312	885.51	0.830	0.830	414.13	0.823	0.004
Protein-energy malnutrition	-15.19	0.783	0.783	25.57	0.290	0.290	2046.99	0.458	0.458	10.06	0.993	0.038
Iodine deficiency							1216.02	0.592	0.592	-356.80	0.715	0.076
Iron-defic. anaemia	2.32	0.898	0.898	-0.98	0.905	0.905	133.93	0.896	0.896	-112.07	0.808	0.002
**Noncommunicable diseases**	3221.80	0.013*	0.013	-1031.20	**0.072**	0.072	9548.75	0.445	0.445	-1388.36	0.804	0.003
**A. Malignant neoplasms**	1106.24	0.015*	0.015	-416.93	0.039*	0.039	9605.19	0.010*	0.010	-3392.94	0.038*	0.029
Mouth, oropharynx cancers	36.03	0.151	0.151	-10.67	0.340	0.340	458.89	0.126	0.126	-140.61	0.292	0.050
Oesophagus cancer	49.52	0.173	0.173	-26.67	0.105	0.105	435.41	0.161	0.161	-229.67	0.102	0.002
Stomach cancer	35.33	0.644	0.644	-44.36	0.201	0.201	304.99	0.584	0.584	-368.76	0.145	0.088
Colon and rectum cancers	177.64	0.024*	0.024	-54.95	0.114	0.114	1563.50	0.012*	0.012	-534.09	**0.051**	0.026
Liver cancer	143.94	0.025*	0.025	-115.37	0.000*	0.000	1540.22	0.020*	0.020	-1209.85	0.000*	0.003
Pancreas cancer	23.32	0.449	0.449	-13.16	0.342	0.342	165.17	0.467	0.467	-89.66	0.380	0.035
Trachea, bronchus, lung cancers	317.74	0.002*	0.002	-128.33	0.005*	0.005	2570.22	0.002*	0.002	-978.02	0.008*	0.015
Melanoma, other skin cancers	35.96	0.009*	0.009	-3.28	0.582	0.582	285.96	0.018*	0.018	-20.61	0.694	0.193
Breast cancer	49.26	0.161	0.161	-0.05	0.997	0.997	718.77	0.037*	0.037	-97.18	0.517	0.260
Cervix uteri cancer	-34.38	0.143	0.143	25.34	0.019*	0.019	-511.32	0.137	0.137	390.70	0.014*	0.217
Corpus uteri cancer	4.58	0.547	0.547	1.71	0.616	0.616	22.83	0.820	0.820	18.53	0.681	0.162
Ovary cancer	17.00	0.123	0.123	-1.70	0.727	0.727	173.75	0.167	0.167	-24.56	0.660	0.304
Prostate cancer	13.16	0.740	0.740	11.42	0.522	0.522	184.43	0.357	0.357	40.41	0.651	0.343
Bladder cancer	48.05	**0.052**	0.052	-10.86	0.318	0.318	331.71	0.173	0.173	-82.05	0.449	0.055
Lymphomas, multiple myeloma	52.83	**0.051**	0.051	-12.66	0.288	0.288	494.94	0.324	0.324	-55.78	0.803	0.022
Leukaemia	40.72	0.040*	0.040	-10.21	0.241	0.241	239.53	0.590	0.590	-24.76	0.901	0.149
**B. Other neoplasms**	-2.62	0.927	0.927	-0.58	0.964	0.964	-56.16	0.866	0.866	23.27	0.876	0.077
**C. Diabetes mellitus**	-52.56	0.640	0.640	36.84	0.467	0.467	-1924.45	0.201	0.201	894.94	0.186	0.022
**D. Endocrine disorders**	9.97	0.732	0.732	-1.54	0.906	0.906	171.04	0.855	0.855	350.54	0.406	0.001
**E. Neuropsychiatric conditions**	139.91	0.312	0.312	-91.98	0.142	0.142	-10094.1	0.010*	0.010	3931.60	0.023*	0.047
Unipolar depressive disorders	-2.03	0.668	0.668	1.49	0.516	0.516	-5743.30	0.008*	0.008	2139.73	0.025*	0.021
Bipolar disorder							-473.79	0.017*	0.017	200.50	0.023*	0.092
Schizophrenia	-3.12	0.374	0.374	1.08	0.535	0.535	-720.33	**0.057**	0.057	225.20	0.180	0.032
Epilepsy	8.46	0.463	0.463	-1.54	0.768	0.768	-739.99	0.031*	0.031	343.99	0.026*	0.173
Alcohol use dis.	-9.51	0.641	0.641	5.02	0.583	0.583	148.18	0.947	0.947	-603.40	0.547	0.059
Alzheimer’s Disease and other dementias	88.88	0.486	0.486	-70.96	0.223	0.223	1953.45	0.037*	0.037	-989.15	0.020*	0.032
Parkinson’s Disease	9.53	0.395	0.395	-2.94	0.564	0.564	603.60	0.005*	0.005	-220.07	0.020*	0.011
Multiple sclerosis	2.01	0.512	0.512	0.90	0.546	0.546	30.68	0.427	0.427	13.06	0.451	0.113
Drug use disorders	8.21	0.673	0.673	-3.21	0.728	0.728	-1158.69	0.318	0.318	654.28	0.211	0.026
Post-traumatic stress disorder							110.89	0.016*	0.016	-52.89	0.011*	0.092
Obsessive-compulsive disorder							-739.14	0.001*	0.001	409.32	0.000*	0.055
Panic disorder							-159.65	**0.065**	0.065	70.83	**0.068**	0.051
Insomnia (primary)							-74.88	0.571	0.571	43.72	0.462	0.145
Migraine							-696.86	**0.059**	0.059	372.64	0.026*	0.044
**F. Sense organ diseases**	-1.49	0.911	0.911	0.33	0.929	0.929	2782.16	0.271	0.271	-2730.55	0.019*	0.000
Glaucoma							273.19	0.312	0.312	-246.13	0.047*	0.033
Cataracts							709.86	0.513	0.513	-755.56	0.126	0.000
Refractive errors							-472.53	0.717	0.717	-326.63	0.577	0.009
Hearing loss							1771.92	**0.064**	0.064	-1048.53	0.016*	0.003
Macular degeneration	-17.09	0.227	0.227	3.15	0.351	0.351	498.59	0.043*	0.043	-353.68	0.002*	0.016
**G. Cardiovascular diseases**	1812.61	0.043*	0.043	-463.67	0.240	0.240	7892.79	0.236	0.236	-1449.94	0.625	0.016
Rheumatic heart dis.	22.92	0.270	0.270	-8.31	0.372	0.372	67.06	0.889	0.889	-20.96	0.922	0.061
Hypertensive heart disease	-13.67	0.910	0.910	28.35	0.601	0.601	-124.58	0.890	0.890	258.89	0.524	0.034
Ischaemic heart dis.	909.98	**0.089**	0.089	-119.30	0.613	0.613	3863.87	0.326	0.326	97.52	0.956	0.008
Cerebrovascular dis.	671.19	0.041*	0.041	-342.99	0.021*	0.021	3301.30	0.104	0.104	-1992.71	0.031*	0.016
Inflammatory heart diseases	52.64	0.160	0.160	-13.68	0.412	0.412	620.14	0.263	0.263	-153.31	0.535	0.003
**H. Respiratory diseases**	273.81	0.171	0.171	-156.44	**0.084**	0.084	4593.75	**0.091**	0.091	-1208.54	0.316	0.032
Chronic obstruct. pulmonary dis.	309.36	**0.096**	0.096	-151.56	**0.071**	0.071	5494.85	0.020*	0.020	-1688.29	0.106	0.026
Asthma	22.89	0.180	0.180	-15.82	0.042*	0.042	-479.61	0.411	0.411	332.56	0.207	0.047
**I. Digestive diseases**	-37.82	0.747	0.747	39.04	0.459	0.459	-385.36	0.828	0.828	583.24	0.466	0.001
Peptic ulcer disease	2.04	0.921	0.921	1.65	0.858	0.858	-43.43	0.908	0.908	26.73	0.874	0.019
Cirrhosis of the liver	-57.16	0.407	0.407	27.19	0.380	0.380	-785.98	0.465	0.465	418.77	0.386	0.007
Appendicitis	-2.45	0.156	0.156	2.08	0.011*	0.011	-45.46	0.113	0.113	36.49	0.006*	0.083
**J. Genitourinary diseases**	-18.19	0.780	0.780	5.86	0.841	0.841	0.47	1.000	1.000	28.95	0.939	0.021
Nephritis, nephrosis	-23.42	0.675	0.675	7.94	0.751	0.751	-282.34	0.594	0.594	157.93	0.507	0.015
Benign prostatic hypertrophy	-2.51	0.445	0.445	1.10	0.457	0.457	-181.50	**0.054**	0.054	42.62	0.304	0.003
**K. Skin diseases**	-23.34	0.302	0.302	15.10	0.153	0.153	-215.20	0.258	0.258	62.62	0.461	0.234
**L. Musculoskeletal diseases**	38.87	0.030*	0.030	-14.29	**0.072**	0.072	1221.92	0.114	0.114	-755.78	0.032*	0.004
Rheumatoid arthritis	-2.43	0.524	0.524	1.77	0.307	0.307	-504.05	0.007*	0.007	282.31	0.001*	0.002
Osteoarthritis	-1.00	0.759	0.759	0.87	0.583	0.583	1514.05	0.019*	0.019	-841.91	0.004*	0.011
**M. Congenital anomalies**	-36.87	0.379	0.379	23.63	0.211	0.211	-2618.25	0.204	0.204	1607.50	**0.085**	0.010
**N. Oral conditions**	0.64	0.304	0.304	-0.13	0.487	0.487	-1510.95	0.001*	0.001	700.93	0.000*	0.000
Dental caries							-1091.04	0.001*	0.001	571.72	0.000*	0.003
Periodontal disease							-20.37	**0.059**	0.059	7.47	0.120	0.081
Edentulism							-405.58	0.012*	0.012	123.74	**0.079**	0.004
**Injuries**	632.81	0.317	0.317	-239.35	0.398	0.398	20934.32	0.459	0.459	-5738.44	0.651	0.013
**A. Unintentional injuries**	232.26	0.426	0.426	-67.92	0.603	0.603	9476.62	0.520	0.520	-1787.56	0.786	0.009
Road traffic accidents	150.45	0.261	0.261	-53.89	0.369	0.369	4820.42	0.349	0.349	-1543.85	0.503	0.051
Poisonings	16.17	0.720	0.720	-1.02	0.960	0.960	554.07	0.574	0.574	-69.86	0.874	0.002
Falls	19.76	0.669	0.669	-15.04	0.470	0.470	1560.03	0.374	0.374	-452.64	0.564	0.051
Fires	-2.06	0.954	0.954	5.58	0.727	0.727	336.76	0.807	0.807	4.64	0.994	0.001
Drownings	18.11	0.353	0.353	-10.86	0.217	0.217	507.47	0.399	0.399	-238.47	0.378	0.077
Other unintentional injuries	25.04	0.839	0.839	10.49	0.850	0.850	1695.05	0.789	0.789	516.85	0.855	0.001
**B. Intentional injuries**	341.57	0.310	0.310	-137.64	0.361	0.361	11464.52	0.430	0.430	-3954.03	0.544	0.016
Self-inflicted injuries	78.61	0.140	0.140	-58.21	0.018*	0.018	1215.72	0.290	0.290	-936.88	**0.074**	0.007
Violence	-13.32	0.783	0.783	13.66	0.531	0.531	-748.67	0.726	0.726	575.46	0.549	0.073
War	659.92	0.618	0.618	-166.09	0.752	0.752	10564.67	0.485	0.485	-4469.09	0.495	0.051

The correlations were estimated with the General Linear Model with GDP per capita, latitude, humidity, medical care expenses and frequencies of smokers in the population as covariates. Positive B corresponds to a positive correlation and negative B to a negative correlation between particular Rhesus factor genotype frequency and the specific disease burden. Significant results (p < 0.05) and trends (p < 0.10) are printed in bold. Asterisks indicate results significant in two-sided tests. p values < 0.0005 are coded as 0.000. No formal correction for multiple tests were done in this exploratory part of the study. The effect size is shown as Eta^2^.

## Discussion

The results of the ecological regression analysis were in a perfect agreement with all three *a priori* hypotheses. They showed that both the frequencies of Rhesus negative homozygotes as well as those of the Rhesus positive heterozygotes correlated (mostly in the opposite direction) with specific disease burdens. The general pattern was that the countries with a high frequency of Rhesus negative homozygotes had lower congenital-anomalies-associated burden and neuropsychiatric condition-associated burden (except Alzheimer’s and Parkinson’s Disease burden) as well as higher cardiovascular and especially malignant neoplasm-associated burden. Noticeably, the only form of cancer expressing a clear opposite trend was cervix uteri cancer, i.e. cancer of viral origin.

Many (but not all) of the disorders observed to be affected by RhD phenotype, such as cardiovascular diseases, lung cancer, liver cancer, asthma, could be considered as “modern” diseases. It is therefore questionable whether they could shape the geographical distribution of RhD allele in the past. However, the RhD minus allele (the deletion) has probably spread from one geographic location in western Europe also relatively recently, definitively after the colonization of Europe by modern *Homo sapiens sapiens*. It is highly probable that in this time people suffered from liver cancer induced by mycotoxins, from lung cancer, from asthma induced by smoke, and after the loss of the skin melanin also from skin cancer in a similar rate as today’s humans.

The results of the current study agree with observations of worse health status of Rhesus negative subjects reported by earlier case control studies [12,13] or observed in the large cohort study performed on a population of 3,130 subjects [14]. However, the results of the ecological regression and the case-control or cohort studies are difficult to compare. For example, in the case-control and cohort studies, but not the ecologic regression study, the effect of RhD negativity on the health status is seemingly increased by an opposite effect of RhD heterozygosity on RhD positive controls consisting of both RhD positive heterozygotes and homozygotes. Moreover, a positive correlation between the focal disease burden and the frequency of a particular RhD genotype observed in an ecological regression study could either be due to an increased sensitivity of Rhesus negative individuals toward the focal disease or a relatively higher resistance or tolerance of these subjects toward other diseases. For example, the protective effect of RhD negativity against many neuropsychiatric disorders observed in the present ecological study could be caused by the fact that RhD negative subjects usually die at an earlier age due to their higher susceptibility to cardiovascular diseases.

It is not clear whether the presence of RhD minus allele alone, or the presence of other alleles in a strong genetic linkage with this allele is responsible for the observed protective effect of heterozygosity. For example, in the Czech population about 95% of subjects with D+C+c-E-e+ phenotype are RhD+ homozygotes and only 4.5 RhD+ heterozygotes (Daniels 2002). Alternative models for explaining observed associations between RhD phenotype and specific disease burdens based on genetic disequilibrium and gene flow (human history) should be tested in future studies.

Limitations of present study: Onset of disorders associated negatively with the frequency of RhD positive heterozygotes, e.g. liver and lung cancer, is rather high in modern human. It is not clear whether decrease of incidence of such disorders could increase fitness of carriers of this phenotype strongly enough. It must be noted, however, that the results of a previous more sensitive case control study performed on a mostly young population indicate that in addition the incidence of many early-age onset disorders, such as diarrhea, thyroiditis, anemia, panic disorder, and scoliosis, was lower in RhD positive subjects.

The interpretations of ecological regression studies are sometimes complicated, especially if aggregated data are used for the estimation of the strength and direction of the influence of particular factors within a population [24,25]. Therefore, one must be very careful with the interpretation of the observed associations between a particular disease burden and the frequency of Rhesus negative subjects within a population. For example, a positive correlation could be due to an increased sensitivity of Rhesus negative individuals to the focal disease or just to higher resistance or tolerance of these subjects to other diseases. Therefore, future studies of the mechanisms of effects of the Rhesus phenotype on risks of particular diseases must be grounded within individual based (case-control and cohort) studies. However, the objective of the present ecological study was to test the heterozygote advantage hypothesis of maintaining the genetic polymorphism in the RHD gene, resulting in polymorphism of the Rhesus factor phenotypes. Based on this hypothesis, I suggested that the frequencies of Rhesus negative homozygotes as well as Rhesus positive heterozygotes should correlate with certain disease burdens and the direction of such correlation will be the opposite for the two phenotypes. The results of present regression study have confirmed these two predictions.

The frequencies of Rhesus positive heterozygotes were calculated from the frequencies of Rhesus negative homozygotes using the Hardy-Weinberg equation. If the conclusions of the present study are correct, then these theoretical frequencies are influenced by a selection against RhD negative homozygotes and in favor of RhD heterozygotes in middle-age and especially in high-age strata. However, H-W equilibrium is being re-established in every generation and the differences between theoretical and real frequencies for reproductive age-strata are probably relatively low, especially in developed countries.

## Conclusions

The study confirmed all three a priori hypotheses: The frequency of Rh negative homozygotes in particular countries correlates (mostly positively) with the incidence of some health disorder in these countries 2) The frequency of Rh positive heterozygotes in particular countries correlates (mostly negatively) with the incidence of some health disorder in these countries 3) The direction of the relation of incidence of the health disorder with frequency of Rh negative homozygotes and with Rh positive heterozygotes are mostly (actually always) the opposite.

Some of the associations observed in the study were relatively strong. For example, the slopes values (*B*) of 1,812 and -463 for cardiovascular diseases suggests that a 1% increase within the frequencies of Rhesus negative homozygotes and Rhesus positive heterozygotes would result in 1,812 more and 463 less cardiovascular disease associated deaths per 100,000 inhabitants, respectively. A positive nonlinear correlation between the frequencies of Rhesus negative homozygotes and Rhesus positive heterozygotes exists in the population equilibrium. Therefore, an increase of the frequency of Rhesus negative homozygotes is always accompanied by an increase of the frequency of heterozygotes within a population. The disadvantage of an increased frequency of Rhesus negative homozygotes in a population is therefore usually at least partly compensated for through an increased frequency of heterozygotes. However, from the point of view of human medicine and especially that of an RhD negative individual, the increased risk of a particular disease associated with one genotype is not compensated for through the decreased risk of a disease in individuals with another genotype.

From the point of view of basic science, the most important merit of this study is its robust support of the heterozygote advantage hypothesis. The results suggest that the Rhesus factor polymorphism is maintained in human populations due to a higher resistance or tolerance of heterozygotes against specific diseases. It could be speculated to what extent the highly uneven distributions of RHD minus alleles in world populations might be the result of a founder event and a gene flow [26] and to what extent it is also modulated by specific selection pressures caused by differences in the geographical distribution of a disease or diseases.

## Supporting Information

S1 Data Data file containing frequencies if RhD genotypes, specific disease burdens and confounding variables for 65 countries. (XLSX)
